# Adherence to the Nordic food-based dietary guidelines in subgroups of the Finnish adult population

**DOI:** 10.29219/fnr.v70.13737

**Published:** 2026-08-11

**Authors:** Tiina Suikki, Heli Tapanainen, Mirkka Maukonen, Satu Männistö, Niina E. Kaartinen

**Affiliations:** 1 Finnish Institute for Health and Welfare, Department of Public Health, Helsinki, Finland; 2 University of Helsinki, Department of Public Health, Helsinkin, Finland; 3 Folkhälsan Research Center, Public Health Research Program, Helsinki, Finland

**Keywords:** adults, food-based dietary guidelines, general population, National Cancer Institute method, nutrition recommendations, usual intake modelling

## Abstract

**Background:**

The most recent Nordic Nutrition Recommendations including food-based dietary guidelines (FBDGs) were published in 2023. The FBDGs covered 15 food groups with seven quantitative recommendations for plant-based and animal-based food groups.

**Objective:**

Our aim was to model usual intake distributions of these food groups and examine adherence to the quantitative recommendations in Finnish men and women as well as across different subgroups.

**Design:**

We used data from the National FinDiet 2017 Survey including 780 men and 875 women aged 18–74 years. Diet was assessed with two non-consecutive 24-h recalls. Usual intake distributions of food groups with quantitative recommendations (whole grains, vegetables, fruits, and berries, nuts and seeds, vegetable oils, red and processed meat, milk and dairy products, and fish and seafood) were modelled with the National Cancer Institute (NCI) method. Based on modelled distributions, proportions of men and women meeting the recommendations for these food groups were assessed by subgroups (age, education, physical activity, weight status, and total energy intake). Statistically significant differences were evaluated with non-overlapping 95% confidence intervals.

**Results:**

In general, men and women fell below the recommendations of plant-based food groups and exceeded the recommendations for animal-based food groups. Older age, higher education level and higher physical activity were associated with higher adherence to the FBDGs. Higher energy intake was associated with higher adherence to plant-based food recommendations and lower adherence to animal-based food recommendations (except for fish and fish products).

**Conclusions:**

In general, Finnish adults are far from meeting the Nordic FBDGs, which highlights the importance of efficient implementation strategies across different population subgroups to increase the consumption of plant-based foods and decrease the consumption of animal-based foods.

## Popular scientific summary

- Usual intake modelling methodology is a robust approach to addressing random error that characterises short-term dietary assessment methods.
- This study provided valuable and precise information on how the population adhered to nutrition recommendations, supporting public health and nutrition policy.
- The results suggested low adherence to the food-based dietary guidelines in the Finnish adult population.

An overall healthy diet is one of the most important factors influencing population health (1). Assessing the food consumption and nutrient intakes at the population level in relation to evidence-based nutrition recommendations, such as food-based dietary guidelines (FBDGs) and dietary reference values, is essential for identifying risk groups, making nutritional policy decisions and focusing behaviour-change strategies (2).

Although human health is at the core of nutrition recommendations and FBDGs, it is increasingly evident that environmental health and sustainability are strongly linked to food production and consumption. Higher adherence to FBDGs has been shown to benefit both human health and environmental sustainability (3). Since the previous edition of the Nordic nutrition recommendations (NNR) from 2012 the need to integrate environmental sustainability into FBDGs to support the achievement of the United Nations’ 17 Sustainable Development Goals has been increasingly recognised (4, 5). In the recent edition, NNR 2023, environmental impacts of food consumption and related food systems were assessed more comprehensively and considered in addition to the health-oriented FBDGs (6). NNR 2023 advocates predominantly plant-based diets with moderate intake of low-fat dairy products and limited amounts of red and processed meat (6).

Assessing proportions of population to adhere to the nutrition recommendations, particularly FBDGs, often requires data on absolute intakes obtained from food records or dietary recalls, which allow comparison of actual food consumption with FBDGs. However, while short-term dietary assessment methods may be suitable for estimating the average dietary intake at the population level, random error caused by within-person variation typically distorts the data and limits the ability to reliably estimate the proportion of the population whose consumption meets, falls below, or exceeds dietary recommendations (7–9). The effect of random error can be corrected with repeated measures of dietary intake or by statistical usual intake modelling, such as Statistical Program to Assess Dietary Exposure (SPADE) or National Cancer Institute (NCI) method (8, 9).

Although usual intake modelling has been increasingly applied in population-based dietary studies in some countries (e.g. the United States, Belgium, Brazil, the Netherlands, Canada, and Switzerland), its use remains limited in many regions (10–18). In the Nordic region, Finland is among the first to apply such methods to assess adherence to FBDGs. The SPADE method has previously been applied to model primarily nutrient intakes and consumption of some foods in the Finnish dietary survey (FinDiet 2017) in relation to the previous national nutrition recommendations from 2014 (2, 19). However, food consumption data have not yet been comprehensively modelled using usual intake methods and compared to the NNR 2023 FBDGs in Finnish adult population subgroups. Evaluating adherence to dietary recommendations across subgroups supports the design of more equitable and effective nutrition policies, acknowledging that uniform strategies may not address the needs of all subgroups.

The FBDGs in the NNR 2023 include seven quantitative recommendations for food groups (e.g. at least 90 grams of whole grains per day) and eight descriptive recommendations (e.g. ‘Limited consumption of sweets and other sugary foods is recommended’.) (6). Adherence to these quantitative food group recommendations has not previously been assessed in Nordic populations. Therefore, we aimed to model usual intakes of seven food groups with quantitative recommendations (whole grains, vegetables, fruits, and berries, nuts and seeds, vegetable oils, red and processed meat, milk and dairy products, and fish and seafood) using the NCI method (9) and to analyse adherence to their targets across subgroups of Finnish men and women. These findings will support the implementation of the NNR 2023 and the new national nutrition recommendations published in 2024 based on the NNR 2023 in the Finnish adult population (20, 21).

## Methods

### Study population

We included participants from the National FinDiet 2017 Survey, the latest in a series of FinDiet surveys conducted since 1982, which was carried out as a sub-study of the comprehensive population-based national FinHealth 2017 Study including questionnaires, anthropometric measures, and blood and urine sample collection (19, 22). The representative FinHealth 2017 sample comprised 10,247 adults aged ≥ 18 years from 50 study sites in mainland Finland selected from the Finnish Population information system using one- and two-stage stratified sampling design (22). Of this sample, 30 % (*n* = 3,099, aged 18–74 years) was selected to the FinDiet 2017 subsample, and 59% (*n* = 1,828) of them participated in the health examination and were eligible for the dietary data collection (19, 23). Participants were asked to attend two non-consecutive 24-h dietary recalls (24 h recall): the first interview between January and May 2017 face-to-face in conjunction with the health examinations, and the second interview via telephone from February to October 2017 with a minimum interval of 8 days between recalls. In total, 1,655 (780 men and 875 women, participation rate 53 % from initial sample) participants were included in the final survey data as 29 participants did not attend the first face-to-face interview, 114 participants were not reached for the second dietary recall, and 16 participants were excluded due to one or two incomplete dietary recalls.

Both studies were conducted according to the guidelines of the Declaration of Helsinki, and all research protocols were approved by the Coordinating Ethics Committee at the Hospital District of Helsinki and Uusimaa (Reference 37/13/03/00/2016). All participants signed the informed consent.

### Dietary assessment

Dietary intake was assessed using two 24 h recalls conducted by trained dietary interviewers according to the EU Menu methodology (23, 24). Portion sizes were estimated with the aid of a validated picture booklet (25, 26), plate models, and examples of standard weights of commercial packaged foods. Dietary information was recorded during the interviews using the in-house dietary software FINESSI (version 5.0.5, Finnish Institute for Health and Welfare, Helsinki, Finland), which utilises the Finnish food composition database, Fineli^®^ (27). Then, consumed dishes were disaggregated into ingredients according to the standard recipes in the Fineli^®^ (except processed meat and fish products that were quantified as purchased) and grouped together based on the Fineli^®^ food grouping system to calculate average daily food consumption and nutrient and energy intake in the Finessi software (28). Next, we composed all plant-based food groups (whole grains, vegetables, fruits, and berries, vegetable oils, nuts and seeds) and animal-based food groups (red and processed meat, milk and dairy products, and fish and seafood) for which quantitative recommendation on daily or weekly consumption were given in the FBDGs in NNR 2023 (Table 1).

**Table 1:** Nordic Nutrition Recommendations 2023 (NNR 2023) food groups with quantitative recommendations (g/day) and food ingredients included

NNR 2023 food groups	Recommendation, g/day	Food ingredient groups included in our analyses^1^
Whole grains	≥ 90	Rye, oat, barley^2^
Vegetables, fruit, and berries	500–800	All vegetables, root vegetables, fruits, and berries *Excluded: potatoes, legumes, and fruit juices*
Nuts and seeds	20–30	All nuts and seeds
Vegetable oils	≥ 25	Plant-based oils, margarines
Red and processed meat	≤ 50^3^	Red meat: beef, pork, lamb, and game as raw weight converted with factor 0.7 to represent cooked weight Processed meat: meat products, cold cuts, sausages^4^
Milk and dairy products	350–500	Dairy products converted as milk equivalents^5^: all milk × 1 yoghurt × 1 cream and ice cream × 2.7 cheese × 5 butter × 6.5
Fish and seafood	43–64^6^	All fish and seafood

1 Consumed foods collected with two non-consecutive 24 h dietary recalls were disaggregated into ingredients according to the standard recipes in the Fineli^®^ (except processed meat and fish products that were quantified as purchased) and grouped together based on the Fineli^®^ food grouping system (27).

2 The combined intake of rye, oat and barley was used to reflect the whole grain intakes (29).

3 Original recommendation of 350 g/week converted into daily consumption recommendation by dividing weekly recommendation by seven.

4 Processed meat was analysed together with red meat, although the NNR 2023 provided only the descriptive recommendation (‘as low as possible’) for processed meat.

5 Conversion factors were based on the Stockholm resilience centre (30).

6 Original recommendation of 300–450 g/week converted into daily consumption recommendation by dividing given range by seven.

### Population subgroups

Age and sex were derived from the sampling frame, while other background variables were obtained from self-administered questionnaires (education and leisure time physical activity) or from the health examination measurements (height and weight). Participants were categorised into three age groups: 18–44, 45–65, and 66–74 years. Education level (low, middle, high) was based on total years of education, which were divided into tertiles by sex and birth year to account for the extension of the basic education system and the increase in average school years over time. Leisure time physical activity was assessed with a three-level scale: inactive (light activities such as reading and watching television), moderately active (walking, gardening, or other activities ≥ 4 h/week), and active (running, swimming or other physically demanding activities ≥ 3 h/week or heavy sports several times/week). Height and weight were measured by trained study nurses at the study sites according to standardised international protocols (31). Body mass index (BMI) was calculated as weight (kg) divided by the height squared (m^2^) and categorised into three categories: BMI: 18.5–24.9 kg/m^2^, 25.0–29.9 kg/m^2^, and ≥ 30 kg/m^2^ (32). For energy categorization, participants by sex were divided into three groups of total energy intake based on quartiles: ‘lowest’ (1^st^ quartile), ‘medium’ (2^nd^ and 3^rd^ quartiles combined), and ‘highest (4^th^ quartile).

### Statistical analyses

Men and women were analysed separately as food consumption and energy intake differ by sex in the Finnish adult population (19). Survey weights were applied to correct for non-response bias and enhance the representativeness of the findings to the Finnish adult population (33). Statistical analyses were conducted with SAS Enterprise Guide software, version 8.5 of the SAS System for Windows.

Baseline characteristics of the participants were calculated as means with standard deviations for continuous variables and as proportions for categorical variables without applying survey weights (Table 2). The NCI method developed by the NCI in the United States (9, 34, 35) was used to estimate usual intake distributions of food groups by different population groups (sex, age, education, BMI, and total energy intake quartiles). The method was also used to assess the proportions of population falling below, meeting or exceeding the given dietary recommendations (Table 1). The bootstrap method with 500 iterations was used to compute the 95% confidence intervals (CI) for each population proportion. Statistical difference between population subgroups for each food group were evaluated by non-overlapping 95% CI (36, 37). Usual intake modelling for nuts and seeds within sex-specific subgroups could not be performed due to limited number of consumption days and low consumption level. The usual intake analyses were carried out using MIXTRAN and DISTRIB macros. The model type ‘amount’ was used for food groups consumed nearly every day: 1) vegetables, fruit, and berries, 2) vegetable oils and 3) milk and dairy products. For episodically consumed foods, either model type ‘corr’ (food groups: 1) whole grains for women, 2) red and processed meat and 3) fish and seafood for men) or ‘nocorr’ (food groups: 1) whole grains for men and 2) fish and seafood for women) was used. Age groups (18–44 years, 45–65 years, and 66–74 years) were included in the model as covariate dummy variables for all subgroup analyses.

**Table 2:** Characteristics of participants in the FinDiet 2017 Survey

	Men	Women
*n*	780	875
Age, years	51 (16)	51 (16)
Educational level, %^1^		
High	33	33
Medium	33	36
Low	34	31
Leisure-time physical activity, %^2^		
Active	33	28
Moderately active	43	47
Inactive	24	25
Weight status		
Weight, kg	86.8 (15.2)	73.2 (15.5)
Height, cm	177.2 (6.8)	163.6 (6.2)
BMI, kg/m^2^	27.6 (4.5)	27.3 (5.6)
BMI 18.5–24.9, %	31	40
BMI 25.0–29.9, %	44	33
BMI ≥ 30.0, %	25	27
Energy intake, MJ	9.0 (2.7)	7.3 (2.0)

Data are presented as means with standard deviations (SD) for continuous variables or percentages for categorical variables.

1 Tertiles of self-reported total school years according to birth cohort to adjust for the extension of the basic education system and the increase of average school years over time; *n*_men_ = 773, *n*_women_ = 859.

2 Self–reported leisure–time physical activity: inactive (light activities such as reading), moderately active (walking/cycling at least 4 h/week), active (physically demanding activities at least 3 h/week); *n*_men_ = 776 *n*_women_ = 867.

## Results

In total, our data sample included 780 (47%) men and 875 (53%) women with mean age 51 (±16) years in both groups (Table 2). The highest proportion of men and women was moderately active on their leisure-time. The mean BMI for men and women were 27.6 and 27.3 kg/m^2^, respectively. The mean daily energy intake was 9.1 (±2.7) MJ/day for men and 7.3 (±2.0) MJ/day for women.

### Usual intakes of plant-based foods and adherence to their recommendations in population subgroups

In general, the mean daily consumption of plant-based foods and the adherence to their recommendations were relatively low in men and women. The majority of men and women fell below the recommendations of plant-based foods; proportion ranging from 66% for vegetable oils (men) to 96% for whole grains (women) (Table 3).

**Table 3:** Usual intake modelled mean consumption of food groups included in the food-based dietary guidelines (FBDG) in the Nordic Nutrition Recommendations 2023 and proportions of Finnish men and women falling below, meeting, or exceeding FBDG quantitative recommendations

Food group	FBDG limits, g/day	Men, *n* = 780		Women, *n* = 875	
		Mean consumption g/day [95% CI]	Proportion of population falling below, meeting, or exceeding the recommendation, % [95% CI]	Mean consumption g/day [95% CI]	Proportion of population falling below, meeting, or exceeding the recommendation, % [95% CI]
Whole grains	< 90	**56** **[52, 61]**	89 [85, 94]	**43** **[40, 48]**	96 [93, 98]
	**≥ 90**		**11 [6, 15]**		**4 [2, 7]**
Vegetables, fruits, and berries	< 500	**301** **[281, 323]**	88 [85, 91]	**362** **[339, 387]**	81 [75, 86]
	**500–800**		**11** [**8.2, 13.7]**		**18 [13.7, 21.2]**
	> 800		1 [0, 2]		1 [1, 3]
Vegetable oils	< 25	**22** **[20, 25]**	**66 [61, 71]**	**15** **[14, 16]**	**86 [83, 89]**
	**≥ 25**		**34 [29, 39]**		**14 [11, 17]**
Red and processed meat	**≤ 50**	**112** **[103, 121]**	**7 [4, 11]**	**62** **[57, 67]**	**31 [23, 38]**
	> 50		**93** [**89, 96]**		**69 [62, 77]**
Milk and dairy products as milk equivalents^1^	< 350	**818** **[767, 869]**	**7 [4.2, 9.1]**	**639** **[604, 673]**	**14 [9.2, 19.4]**
	**350–500**		**12 [10, 14]**		**20 [18, 21]**
	> 500		**81 [77, 86]**		**66 [60, 73]**
Fish and seafood	< 43	34 [29, 40]	72 [63, 80]	26 [22, 31]	87 [77, 99]
	**43–64**		**16 [13, 18]**		**11 [1, 17]**
	> 64		**12 [7, 19]**		**2 [0, 6]**

Grey boxes indicate recommendation for each food group.

Difference between mean consumption of food groups and proportions of men and women falling below, meeting, or exceeding the recommendations were analysed with non-overlapping 95% CIs, bolded numbers indicating statistically significance difference.

1 Dairy products were converted to represent liquid milk with converting factors based on the Stockholm resilience centre (30)

**Fig. 1:**
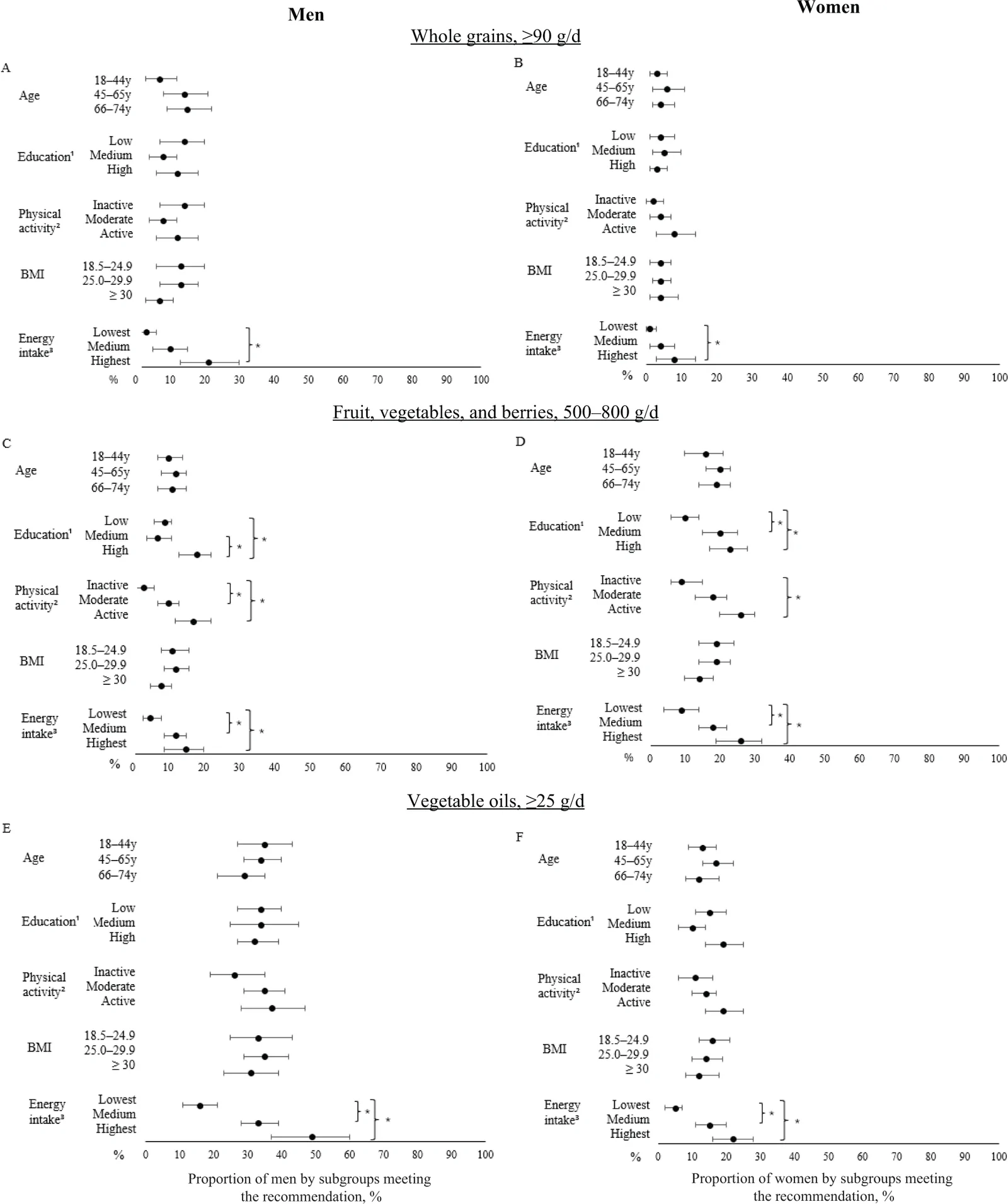
Proportion of men (a, c, e) and women (b, d, f) by different subgroups (age, education, physical activity, BMI, and energy intake) meeting the recommendation for plant-based food groups: whole grains ≥ 90 g/day (a, b), fruit, vegetables, and berries 500–800 g/day (c, d), and vegetable oils ≥ 25 g/day (e, f). Difference between proportions within subgroups analysed with non-overlapping 95% CIs, asterisks indicating statistically significant difference within groups. ^1^Tertiles of self-reported total school years according to birth cohort to adjust for the extension of the basic education system and the increase of average school years over time. ^2^Self-reported leisure–time physical activity: inactive (light activities such as reading), moderately active (walking/cycling at least 4 h/week), active (physically demanding activities at least 3 h/week). ^3^Quartiles of total energy intake: lowest indicating the first quartile, medium indicating second and third quartiles combined, highest indicating the fourth quartile.

In more detail, men consumed more whole grains compared to women (56 g/day [95% CI: 52, 61] and 43 g/day [40, 48], respectively) (Table 3). Moreover, differences in adherence to the recommendation (≥ 90 g/day) were observed only between energy-intake subgroups, with participants in the highest energy-intake group being more likely to meet the recommendation than those in the lowest group: (in men: 21% [13, 30] and 3% [1, 6], respectively; in women: 8% [4, 14] and 1% [0, 3], respectively) (Fig. 1a, b, Supplementary Tables 1 and 2).

The mean consumption of vegetables, fruit, and berries was lower in men compared to women (301 g/day [281, 323] and 361 g/day [339, 387], respectively) and women tended to meet the recommendation for this food group (500–800 g/day) more often compared to men (18% [13.7, 21.2] and 11% [8.2, 13.7], respectively). In more detail, men and women in the highest education and physically active groups met the recommendation more often than those in the lowest groups (highest vs. lowest education in men: 18% [13, 22] and 9% [6, 11], in women: 23% [17, 28] and 10% [6, 14]: physically active vs. inactive in men: 17% [12, 22] and 3% [1, 6], in women: 26% [20, 30] and 9% [6, 15]) (Fig. 1c, d, Supplementary Tables 1 and 2). Furthermore, both men and women in the highest energy intake group were more likely to meet the recommendation of vegetables, fruits and berries (highest vs. lowest energy intake groups in men: 15% [9, 20] and 5% [3, 8], in women: 26% [19, 32] and 9% [4, 14]) (Fig. 1c, d, Supplementary Tables 1 and 2).

The mean consumption of vegetable oils was higher in men compared to women (22 g/day [20, 25] and 15 g/day [14, 16], respectively) and men also met the recommendation (≥ 25 g/day) more often compared to women (34% [29, 39] and 14% [11, 17], respectively) (Table 3). Again, men and women in the highest energy intake group met the recommendation for vegetable oils more often compared to the participants in the lowest group (highest vs. lowest energy intake groups in men: 49% [37, 60] and 16% [11, 21], in women 22% [16, 28] and 5% [2, 7]) (Fig. 1e, f, Supplementary Tables 1 and 2).

### Usual intakes of animal-based foods and adherence to their recommendations in population subgroups

Overall, the mean daily consumption of animal-based foods exceeded the recommendations in both men and women, except for fish and seafood, for which the average consumption was below the recommendation. Furthermore, men were more likely than women to exceed, rather than meet, the recommendations for animal-based food groups (Table 3).

**Fig. 2:**
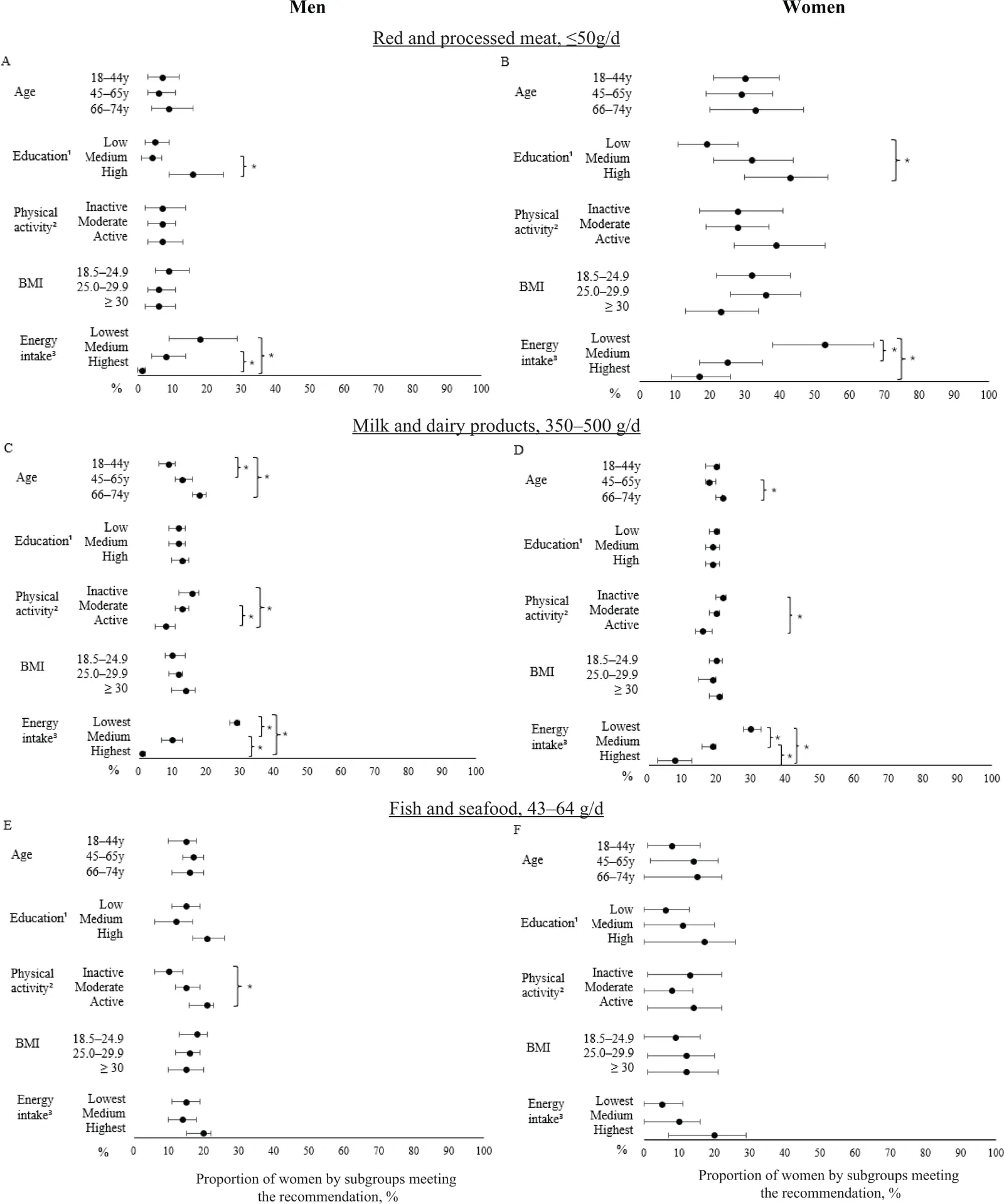
Proportion of men (a, c, e) and women (b, d, f) by different subgroups (age, education, physical activity, BMI, and energy intake) meeting the recommendation for animal-based food groups: red and processed meat ≤ 50 g/day (a, b), milk and dairy products 350–500 g/day (c, d), and fish and shellfish 43–64 g/day (e, f). Difference between proportions within subgroups analysed with non-overlapping 95% CIs, asterisks indicating statistically significant difference within groups. ^1^Tertiles of self-reported total school years according to birth cohort to adjust for the extension of the basic education system and the increase of average school years over time. ^2^Self-reported leisure-time physical activity: inactive (light activities such as reading), moderately active (walking/cycling at least 4 h/week), active (physically demanding activities at least 3 h/week). ^3^Quartiles of total energy intake: lowest indicating the first quartile, medium indicating second, and third quartiles combined, highest indicating the fourth quartile.

Men consumed almost two times more red and processed meat compared to women (112 g/day [103, 121] and 62 g/day [57, 67], respectively) and over four times higher proportion of women met the recommendation for this food group (≤ 50 g/day) compared to men (31% [23, 38] and 7% [4, 11], respectively) (Table 3). Furthermore, in men, the recommendation for red and processed meat was more frequently met in the highest educational group compared with the middle group (16% [9, 25] and 4% [1, 7], respectively) (Fig. 2a, Supplementary Table 1). In women, the same applied for the comparison between the highest and the lowest educational group (43% [30, 54] and 19% [11, 28]), respectively) (Fig. 2b, Supplementary Table 2). In both sexes, those with the lowest energy intake met the recommendation more often than those with the highest intake (lowest vs. highest energy intake groups in men: 18% [9, 29] and 1% [0, 2], in women 53% [38, 67] and 17% [9, 26]) (Fig. 2a, b, Supplementary Tables 1 and 2).

The mean daily consumption of milk and dairy products was higher in men compared to women (818 g/day [767, 869] and 639 g/day [604, 673], respectively); yet, women were two times more likely than men to meet the recommendation for this food group (350–500 g/day) (20% [18, 21] and 11% [10, 14], respectively) (Table 3). Men and women in the oldest age group and in the lowest physical activity group met the recommendation for this food group more often compared to their counterparts in the youngest (in men) or middle (in women) age groups and the group with highest physical activity (oldest vs. youngest groups in men: 18% [16, 20] and 9% [6, 11]; oldest vs. middle aged groups in women: 22% [20.3, 22.5] and 18% [16.6, 19.5]; inactive vs. active in men: 16% [12, 18] and 8% [5, 11], in women: 22% [20, 23] and 16% [14, 19]) (Fig. 2c, d, Supplementary Tables 1 and 2). Moreover, men and women in the lowest energy intake group met the recommendation for milk and dairy products more often compared to their counterparts in the highest group (lowest vs. highest energy intake groups in men: 29% [27, 30] and 1% [0, 2], in women: 30% [28, 33] and 8% [3, 13]) (Fig. 2c, d, Supplementary Tables 1 and 2).

The mean daily consumption of fish and seafood did not differ statistically between men and women (34 g/day [29, 40] and 26 [22, 31] g/day, respectively) but no difference in adherence to the daily recommendation (43–64 g/day) between sexes was detected (Table 3). The only difference between population subgroups in adherence to the fish and seafood recommendation was detected between men in the physically active and inactive groups (21% [16, 23] and 10% [6, 14], respectively) (Fig. 2e, Supplementary Table 1).

## Discussion

Our study is among the first to comprehensively examine the adherence to the quantitative FBDGs included in the NNR 2023 using usual intake modelling methodology. Our results revealed generally low adherence to these updated guidelines in Finnish adults. Especially, a high proportion of the population fell below the recommendations for plant-based foods and exceeded the recommendations for animal-based foods, notably for red and processed meat as well as milk and dairy products. In general, women were more likely than men to meet the recommendations. Furthermore, the participants’ age, education level, leisure-time physical activity, and total energy intake were associated with adherence to certain FBDGs.

Globally, adherence to the FBDGs assessed by using usual intake modelling methods has been found to be generally low (10–18). For instance, 5% in the general Belgian population (*n* = 3,146, 3–64 years) in 2014 met the recommendation for vegetables (100–300 g/day) and 9% met the recommendation for fruits (excluding fruit juices, 100–375 g/day) (11). Furthermore, 26% in Canadian general population aged 2 years and older (*n* = 17,509) met the minimum number of daily age- and sex-specific recommendations varying from 7 to 10 servings in 2004 (13). Similarly, only 13% of US men and women (≥ 19 years, n_men_ = 4,381, n_women_ = 4,745) met the recommendation for total vegetables and 15% of men and 20% of women met the recommendations for total fruit (2 cup-equivalents for men and 1.5 for women) in 2001−2004 (12). Whereas, in the present study, 11% of men and 17% of women achieved the 500–800 g/day recommendation. In contrast, in Switzerland (*n* = 2,057, 18–75 years) higher proportions of men (56%) and women (70%) met the recommendations for red meat (≤ 35 g/day of cooked meat, excluding processed meat) in 2014 (14) compared to the proportions of Finnish men (7%) and Finnish women (31%) meeting the recommendation for red and processed meat (≤ 50 g/day of cooked meat) in the NNR 2023. Moreover, in the Nordic countries, usual intake methods have not been used for modelling food consumption distributions, although differences in the average daily food consumption across Nordic and Baltic countries have been reported in a background article for the NNR 2023 (38). Overall, variation in food group definitions, dietary recommendations, and the age composition of study populations limits reliable comparisons between countries.

### Compliance with FBDGs by population subgroups

In our study, women were more likely than men to meet the recommendations, especially for red and processed meat and vegetables, fruit, and berries. These findings are in accordance with previous studies (10, 11, 18, 19). This could be attributable to women’s tendency to be more healthoriented and may experience greater social pressure regarding dietary choices. In contrast, men often participate less in food and nutrition related household activities, which may contribute to lower nutrition knowledge and different health beliefs (39–43).

Furthermore, age could influence food consumption and impact the likelihood of meeting FBDGs. For example with aging, appetite reduces due to increasing levels of satiety-related hormones such as insulin, leptin, cholecystokinin and peptide-YY leading to lower food consumption and nutrient and energy intake (44, 45). In addition, aging often introduces changes in the social environment such as living alone and decreased interactions with others, thereby affecting dietary habits (46). However, evidence from global populations suggests that the consumption of several healthy foods tends to increase with age (47). Especially consumption of fruit and vegetables increased with age and consumption of red and processed meat decreased. Furthermore, the Dutch population-based food consumption survey (2019–2021) (*n* = 3,570, aged 1–79 years) showed that the proportion of population meeting the guideline for vegetables (200 g/day) increased with age (18). In the current study, no significant differences in vegetable, fruit, and berry consumption were observed between age groups. However, older men consumed more whole grains than younger men, which may reflect a higher consumption of, for example, rye bread and oatmeal porridge (19). Older men also consumed less red and processed meat compared to the youngest men. Moreover, both older men and women were more likely to meet the recommendation for milk and dairy products than their younger counterparts.

A recent narrative review highlighted educational attainment as a super determinant of diet quality, as it not only drives income but also shapes material, cultural, social, and cognitive pathways, including health literacy, occupational conditions, social networks, and long-term orientations, that collectively influence dietary behaviours and inequities across the life course (48). Consistent with this, higher education level has been associated with higher diet quality globally (49, 50). Our results are in accordance with this as both men and women in the highest education group met the recommendation for vegetables, fruit, and berries and red and processed meat more likely compared the lowest educational group.

More physically active lifestyles have been associated with healthier diets attributable to improved eating behaviour (i.e. eating self-regulation) and appetite control (51). However, there is limited evidence about the association between physical activity (as on exposure) and adherence to FBDGs (as on outcome). In our study, the most physically active participants were more likely to meet the recommendations for vegetables, fruits and berries compared with physically inactive participants. This finding is consistent with the finding among Brazilian urban population (*n* = 506, age 40 [standard deviation, SD 15.6] years) (16). However, in our study the physically active men exceeded the recommendations for milk and dairy products more often compared to the inactive counterparts. The higher consumption levels observed in Finnish active participants may reflect both the healthy image of dairy products, particularly their protein content, and the wide availability and diverse selection of such products in the markets in Finland.

Although overall diet quality and consumption of individual food groups have been associated with body size, evidence regarding the association between body size and adherence to the FBDGs is limited (52). In our study, no differences between BMI groups were detected either in mean consumption or in meeting the recommendations across any of the food groups. This finding may be explained by previous evidence suggesting that individuals living with overweight or obesity are more prone to underreport their food consumption or intentionally consume less food (53). Similarly, Silva et al. reported no difference in adherence to the food group recommendations included in the Brazilian national dietary guidelines between BMI categories in Brazilian urban population (*n* = 506, age 40 [SD 15.6] years), except that participants in the highest BMI group were less likely to meet the recommendation for sugar intake compared with those in the two lower BMI groups (16).

### Total energy intake and compliance with FBDGs

Total energy intake is a crucial factor to consider when analysing adherence to the FBDGs as some of the recommendations may be met and some could be exceeded more easily with higher energy intake and therefore higher food consumption. This was evident in the current study, as participants in highest energy intake group met the plant-based recommendations and exceeded the animal-based recommendations more often. Generally men, younger people, participants with more physical activity, and participants living with higher weight require and have higher energy intakes than those with lower body weight (54). In the NNR 2023, the FBDG were set based on daily reference values for normal body weights (BMI: 23 kg/m^2^) and for a physical activity level of 1.6 which corresponds to daily energy requirement of 11.3 MJ/day for men and 9 MJ/day for women, the range depending on the age (6). In the current study, the mean daily energy intake was 9.0 MJ/day for men and 7.3 MJ/day for women being clearly under the recommended levels. Future analyses of adherence to the FBDGs could benefit from assessing adherence using energy-standardised food consumption targets or as proportions of total daily energy intake.

### Strengths and limitations

This study had several strengths and potential limitations. A key strength was that the data collection followed the EFSA EU Menu methodology and was controlled and standardised (19, 23). In addition, to address potential selection bias, population weights were applied to correct for non-participation and to improve the representativeness of consumption estimates for the general adult population, as healthier individuals are typically more likely to participate in health examination surveys (55–57). The potential impact of weekdays versus weekend days and seasonal variation was also considered, as 71% of data collection days were weekdays and 29% weekend days (19). Moreover, the use of two non-consecutive 24 h dietary recalls to assess dietary intake and applying usual intake modelling method to analyse proportions of population meeting the FBDGs were strengths of the study (9).

A limitation of this study is that due to cross-sectional design, causal relationships between dietary intake and population subgroups variables, such as physical activity and weight status, could not be established. Furthermore, all self-reporting methods, including 24 h dietary recalls conducted by well-trained dietary interviewers, may always include some degree of recall bias. Another limitation was an absence of energy consideration when analysing compliance with the FBDGs. However, this limitation was addressed by analysing men and women separately, and by assessing adherence to the FBDGs according to energy intake quartiles. Finally, analysing the adherence to all FBDGs simultaneously could have offered deeper insight into the diet quality in/of Finnish adults. However, owing to the relative novelty of usual-intake methods and methodological constraints in applying them, we were unable to incorporate this analysis within the scope of this study. In general, studies exploring the adherence to several FBDGs simultaneously are scarce and subject to methodological limitations. For example, in a Swiss population-based study (*n* = 2,057, mean age of 46.1 ± 15.4 years) based on two 24 h dietary recalls, 0.1% of the population was found to follow all seven FBDGs included in the study, and about 41% followed at least three recommendations (14). However, in the study, usual intake modelling was not applied, which limits the reliability of the adherence estimates.

## Conclusion

This study described the usual intake modelled distributions of food consumption included in the FBDGs of the NNR 2023 and assessed adherence to these recommendations in Finnish adults. In general, the diets of Finnish men and women remained far from these targets. Consumption of plant-based food groups, that is, whole grains and vegetables, fruits and berries fell consistently below them, whereas consumption of animal-based food groups, that is, milk and dairy products as well as red and processed meat, clearly exceeded the recommendations. Especially, age, education, leisure-time physical activity, and total energy intake were important determinants of FBDGs adherence and should therefore be considered for nutrition policy and behaviour-change interventions. In general, more ambitious political decision and broader cross-sector collaboration are needed to promote more sustainable diets in Finnish adults. For example, food education could strengthen nutrition-related knowledge and skills, while food services, the food industry, and retailers could support consumers in making healthier and more sustainable food choices.

## Supplementary Material


