# Supplementary material for: Adherence to the Nordic food-based dietary guidelines in subgroups of the Finnish adult population
Source: Food Nutr Res. 2026 Aug 11;70:10.29219/fnr.v70.13737. doi: 10.29219/fnr.v70.13737 (PMC13563447; doi:10.29219/fnr.v70.13737)
Supplement: Supplementary file 1 [file FNR-70-13737-s1.docx]

Supplemental table 1. Usual intake modelled mean consumption of food groups included the food-based dietary guidelines (FBDG) in the Nordic Nutrition Recommendations 2023 and proportions of Finnish men by age, education, physical activity, weight status, and energy intake quartiles falling below, meeting, and exceeding the quantitative recommendations.

BMI, body mass index; FBDG, food-based dietary guidelines

Difference between mean consumption of food groups and proportions of men by subgroups falling below, meeting, and exceeding the recommendations were analysed with non-overlapping 95% CIs, bolded numbers indicating statistically significance difference.

^1^Tertiles of self-reported total school years according to birth cohort to adjust for the extension of the basic education system and the increase of average school years over time.

^2^Self-reported leisure-time physical activity: inactive (light activities such as reading), moderately active (walking/cycling at least 4 h/wk), active (physically demanding activities at least 3 h/wk)

^3^Quartiles of total energy intake: lowest indicating the first quartile, medium indicating second, and third quartiles combined, highest indicating the fourth quartile.

^4^Dairy products were converted to represent liquid milk with converting factors based on the erratum of Stockholm resilience centre [<https://www.stockholmresilience.org/download/18.8620dc61698d96b1901719c/1561013818461/Erratum_Nordic%20report_14-6-19.pdf>].

|  |  |  | Age | | |  | Education^1^ | | |  | Physical activity^2^ | | |  | BMI | | |  | Energy intake^3^ | | |
| --- | --- | --- | --- | --- | --- | --- | --- | --- | --- | --- | --- | --- | --- | --- | --- | --- | --- | --- | --- | --- | --- |
| FBDG | |  | 18–44y | 45–65y | 66–74y |  | Low | Middle | High |  | Inactive | Moderate | Active |  | 18.5–24.9  kg/m^2^ | 25.0–30.0  kg/m^2^ | >30  kg/m^2^ |  | Lowest | Medium | Highest |
| **Women, n** |  |  | **311** | **317** | **247** |  | **285** | **305** | **269** |  | **238** | **409** | **220** |  | **348** | **291** | **236** |  | 218 | 438 | 219 |
|  |  |  |  |  |  |  |  |  |  |  |  |  |  |  |  |  |  |  |  |  |  |
| Whole grains | mean, g/d |  | 39 [33,45] | 49 [44,54] | 46 [41,51] |  | 43 [38,48] | 46 [40,52] | 42 [37,48] |  | **36 [30,42]** | 43 [39,48] | **52 [47,59]** |  | 43 [38,48] | 44 [40,49] | 44 [38,50] |  | **33 [28,39]** | **44 [40,49]** | **52 [47,58]** |
|  | <90g, % |  | 97 [94,99] | 94 [89,98] | 96 [92,98] |  | 96 [92,99] | 95 [90,98] | 97 [94,99] |  | 98 [95,100] | 96 [93,99] | 92 [86,97] |  | 96 [93,99] | 96 [93,98] | 96 [91,99] |  | **99 [96.8,99.8]** | 96 [92.4,98.8] | **92 [86.2,96.5]** |
|  | **≥90g, %** |  | 3 [1,6] | 6 [2,11] | 4 [2,8] |  | 4 [1,8] | 5 [2,10] | 3 [1,6] |  | 2 [0,5] | 4 [1,7] | 8 [3,14] |  | 4 [1,7] | 4 [2,7] | 4 [1,9] |  | **1 [0,3]** | 4 [1.2,7.6] | **8 [4,14]** |
|  |  |  |  |  |  |  |  |  |  |  |  |  |  |  |  |  |  |  |  |  |  |
| Vegetables, fruits, and berries | mean, g/d |  | 347 [309,389] | 378 [354,404] | 372 [343,400] |  | **304 [280,335]** | **379 [350,413]** | **403 [365,437]** |  | **303 [271,339]** | 367 [336,397] | **419 [384,455]** |  | 374 [341,408] | 371 [343,403] | 336 [306,368] |  | **295 [259,332]** | **371 [345,397]** | **417 [374,466]** |
|  | <500g, % |  | 83 [76,90] | 78 [73,83] | 79 [73,85] |  | **90 [85,94]** | **78 [72,84]** | **74 [67,82]** |  | **90 [84,94]** | 80 [74,86] | **71 [63,79]** |  | 79 [72,85] | 79 [73,85] | 85 [79,90] |  | **91 [85.1,95.7]** | 80 [74.9,85.2] | **71 [61.4,80.2]** |
|  | **500**–**800g, %** |  | 16 [10,21] | 20 [16,23] | 19 [14,23] |  | **10 [6,14]** | **20 [15,25]** | **23 [17,28]** |  | **9 [6,15]** | 18 [13,22] | **26 [20,30]** |  | 19 [14,24] | 19 [14,23] | 14 [10,18] |  | **9 [4.3,13.7]** | **18 [14.3,22.1]** | **26 [18.9,32.0]** |
|  | >800g, % |  | 1 [0,3] | 2 [1,4] | 2 [1,4] |  | 0 [0.1,1.4] | 2 [0.6,3.6] | 3 [0.9,5.2] |  | 1 [0.1,1.3] | 2 [0.5,3.2] | 3 [1.2,6.3] |  | 2 [1,4] | 2 [1,4] | 1 [0,2] |  | 0 [0.1,1.2] | 2 [0.5,3.0] | 3 [0.9,6.6] |
|  |  |  |  |  |  |  |  |  |  |  |  |  |  |  |  |  |  |  |  |  |  |
| Vegetable oils | mean, g/d |  | 15 [13,16] | 16 [15,18] | 14 [13,17] |  | 16 [14,18] | 13 [12,15] | 17 [15,19] |  | 14 [12,16] | 15 [14,16] | 17 [15,19] |  | 16 [14,18] | 15 [13,17] | 14 [12,17] |  | **11 [9,12]** | **16 [14,17]** | **18 [16,20]** |
|  | **<25g, %** |  | 87 [83,91] | 83 [78,87] | 88 [82,92] |  | 85 [79.7,88.8] | 90 [85.5,93.9] | 81 [75.5,86.3] |  | 89 [84,94] | 86 [83,90] | 81 [75,86] |  | 84 [79,88] | 86 [81,90] | 88 [82,92] |  | **95 [93,98]** | **85 [80,89]** | **78 [72,84]** |
|  | ≥25g, % |  | 13 [9,17] | 17 [13,22] | 12 [8,18] |  | 15 [11,20] | 10 [6,15] | 19 [14,25] |  | 11 [6,16] | 14 [10,17] | 19 [14,25] |  | 16 [12,21] | 14 [10,19] | 12 [8,18] |  | **5 [2,7]** | **15 [11,20]** | **22 [16,28]** |
|  |  |  |  |  |  |  |  |  |  |  |  |  |  |  |  |  |  |  |  |  |  |
| Red and processed meat | mean, g/d |  | 63 [56,71] | 62 [57,69] | 58 [52,65] |  | **71 [64,79]** | 61 [53,69] | **53 [47,59]** |  | 65 [56,73] | 64 [58,71] | 56 [49,63] |  | 61 [55,67] | 58 [52,64] | 68 [59,78] |  | **47 [41,55]** | **63 [57,68]** | **76 [67,86]** |
|  | **≤50g, %** |  | 30 [21,40] | 29 [19,38] | 33 [20,47] |  | **19 [11,28]** | 32 [21,44] | **43 [30,54]** |  | 28 [17,41] | 28 [19,37] | 39 [27,53] |  | 32 [22,43] | 36 [26,46] | 23 [13,34] |  | **53 [38,67]** | **25 [17,35]** | **17 [9,26]** |
|  | >50g, % |  | 70 [60,79] | 71 [62,81] | 67 [53,80] |  | **81 [72,89]** | 68 [56,79] | **57 [46,70]** |  | 72 [59,83] | 72 [63,81] | 61 [47,73] |  | 68 [57,78] | 64 [54,74] | 77 [66,87] |  | **47 [33,62]** | **75 [65,83]** | **83 [74,91]** |
|  |  |  |  |  |  |  |  |  |  |  |  |  |  |  |  |  |  |  |  |  |  |
| Milk and dairy products  as milk equivalents^4^ | mean, g/d |  | 635 [585,688] | 666 [624,706] | 606 [561,654] |  | 627 [576,684] | 637 [579,689] | 649 [606,367] |  | **593 [546,643]** | 631 [580,680] | **707 [648,759]** |  | 634 [590,672] | 664 [612,723] | 623 [575,672] |  | **429 [389,465]** | **660 [628,695]** | **822 [751,890]** |
|  | <350g, % |  | 14 [8,21] | 12 [8,17] | 17 [12,23] |  | 15 [9,22] | 15 [9,22] | 14 [9,19] |  | 18 [12,24] | 15 [9,21] | 9 [6,14] |  | 15 [9,20] | 12 [7,18] | 16 [10,22] |  | **38 [29,47]** | **8 [4,12]** | **2 [0,6]** |
|  | **350**–**500g, %** |  | 20 [17.1,21.2] | **18 [16.6,19.5]** | **22 [20.3,22.5]** |  | 20 [18,21] | 19 [17,21] | 19 [17,21] |  | **22 [20,23]** | 20 [18,21] | **16 [14,19]** |  | 20 [18,22] | 19 [15,20] | 21 [18,22] |  | **30 [28,33]** | **19 [16,20]** | **8 [3,13]** |
|  | >500g, % |  | 66 [58,74] | 70 [64,76] | 61 [55,68] |  | 65 [57,73] | 66 [57,74] | 67 [61,74] |  | 60 [52,68] | 65 [57,73] | 75 [67,81] |  | 65 [58,72] | 69 [62,79] | 63 [56,72] |  | 32 [25,39] | 73 [68,81] | 90 [81,96] |
|  |  |  |  |  |  |  |  |  |  |  |  |  |  |  |  |  |  |  |  |  |  |
| Fish and seafood | mean, g/d |  | 24 [18,31] | 29 [23,35] | 28 [21,36] |  | 22 [17,28] | 26 [20,34] | 32 [25,38] |  | 28 [21,36] | 24 [19,29] | 29 [23,36] |  | 25 [20,30] | 27 [21,34] | 27 [20,35] |  | 20 [14,28] | 26 [21,31] | 33 [26,42] |
|  | <43g, % |  | 91 [80,99] | 84 [71,98] | 84 [68,100] |  | 94 [84,100] | 88 [75,100] | 80 [64,100] |  | 85 [71,99] | 91 [82,100] | 83 [68,99] |  | 89 [79,100] | 86 [73,99] | 86 [72,99] |  | 95 [86,100] | 89 [80,100] | 75 [59,93] |
|  | **43**–**64g, %** |  | 8 [1,16] | 14 [2,21] | 15 [0,22] |  | 6 [0,13] | 11 [0,20] | 17 [0,26] |  | 13 [1,22] | 8 [0,14] | 14 [1,22] |  | 9 [0,16] | 12 [1,20] | 12 [1,21] |  | 5 [0,11] | 10 [0,16] | 20 [7,29] |
|  | >64g, % |  | 1 [0,4] | 2 [0,8] | 1 [0,9] |  | 0 [0,3] | 1 [0,6] | 3 [0,10] |  | 2 [0,7] | 1 [0,4] | 3 [0,9] |  | 1 [0,6] | 2 [0,7] | 2 [0,8] |  | 0 [0,3] | 1 [0,4] | 5 [0,15] |

Supplemental table 2. Usual intake modelled mean consumption of food groups included the food-based dietary guidelines (FBDG) in the Nordic Nutrition Recommendations 2023 and proportions of Finnish women by age, education, physical activity, weight status, and total energy intake falling below, meeting, and exceeding the quantitative recommendations.

BMI, body mass index; FBDG, food-based dietary guidelines

Difference between mean consumption of food groups and proportions of men by subgroups falling below, meeting, and exceeding the recommendations were analysed with non-overlapping 95% CIs, bolded numbers indicating statistically significance difference.

^1^Tertiles of self-reported total school years according to birth cohort to adjust for the extension of the basic education system and the increase of average school years over time.

^2^Self-reported leisure-time physical activity: inactive (light activities such as reading), moderately active (walking/cycling at least 4 h/wk), active (physically demanding activities at least 3 h/wk).

^3^Quartiles of total energy intake: lowest indicating the first quartile, medium indicating second, and third quartiles combined, highest indicating the fourth quartile.

^4^Dairy products were converted to represent liquid milk with converting factors based on the erratum of Stockholm resilience centre [<https://www.stockholmresilience.org/download/18.8620dc61698d96b1901719c/1561013818461/Erratum_Nordic%20report_14-6-19.pdf>].
